# A Syrian Mental Hospital

**Published:** 1924-07

**Authors:** 


### A SYRIAN MENTAL HOSPITAL.

The annual meeting of the Lebanon Mental Hospital at Asfuriyeh, Beyrout, Syria, was held on June 3, at the Caxton Hall, Westminster. The chairman, Dr. E. W. G. Masterman, said that at last the boring for water had been accomplished, and after going 300 feet water had been reached, and had risen considerably in the bore hole. There is now every reason to believe that there will be enough water for the hospital's needs and it has become possible to have a steam laundry, and to develop a home farm, with dairy and vegetable garden, which will go a considerable extent towards making the hospital self-supporting, and reducing the cost of maintenance. The hospital work is on a scientific basis, and Dr. Watson Smith, the Director, hopes that the opportunities there will be used for research and the development of the pathological side and teaching. Already the director is giving instruction to students of the American University in Beyrout, who will in future be the outstanding men of the profession in the Near East. This work is not only philanthropic but educational.

### Leading the Way.

The Rev. E. M. Bickersteth, of the Jerusalem and the East Mission, in moving the adoption of the report, said that he was at Asfuriyeh a little over two months ago and spoke of the importance of the training of students who would be scattered and of the influence the hospital must have over these men. Not only Syria and Palestine, but all the Near East would owe it a debt because it is undoubtedly leading the way in the treatment of mental disease. Mr. Herbert Catford, in seconding, said that he had just visited Beyrout, which in an extraordinary way which no one could have foreseen has become the starting point for the overland road to Bagdad and is now within 10 or 11 hours easy motor drive of Jerusalem. It has the only University in Palestine or Syria, and is now, owing to the increased easiness of transit, getting quite a large number of students from Mesopotamia. The school of Medicine has somewhere about 100 students, and, like Mr. Bickersteth, he was impressed by the importance of Dr. Watson Smith's work in the University, and by the way the hospital has secured the support of the foreign community in Beyrout. Dr. R. Percy Smith moved the adoption of the director's report.

---

### QUEEN'S HOSPITAL FOR CHILDREN.

The Queen's Hospital for Children, in the Hackney Road, Bethnal Green, is comparatively little known in the West End, partly owing to its out-of-the-way position and partly to the fact that its financial resources do not allow it to be much advertised or to maintain a very handsome building. Its very position, however, in a neighbourhood where there is a big population and much poverty, makes it all the more needful of support, yet we learn from the hospital's Fifty-sixth Annual Report that already, before the first half of this year is out, there is a debt of nearly £11,000. At the end of 1923 the total receipts of the hospital were £361 15s. 2d. short of the expenditure, and this, added to £4,031 brought over from 1922, left a deficit of £4,393. The number of in-patients was 1,786, and of out-patients 37,901. The hospital's branch at Bexhill, known as the Little Folks' Home, where there are thirty-six beds, showed a deficit of £688 19s. 6d.

### A Grave Contingency.

At the annual meeting, held on May 29, when the Chair was taken by Colonel Lord William Cecil, C.V.O., it was clearly pointed out—though with much regret—that, in view of this large debt and of the fact that the limit of the hospital's borrowing powers at the bank had been reached, unless a substantial sum of money is assured to the hospital before the end of the year, the committee will be obliged to take that drastic step which it is so important to avoid—the closing down of some portion of the hospital. In 1921 a West End Appeal Office was started in that part of London, but the resulting contributions were found not to justify the expenditure involved. Such an event as the closing down of part of the hospital would be a calamity to the neighbourhood which it serves.

---

The Syllabus for the Training of Nursing Orderlies, "for the information and guidance of medical officers and members of Princess Mary's Royal Air Force Nursing Service engaged in instruction and under instruction," is published for the Air Ministry by His Majesty's Stationery Office. Price, twopence.

